# Beyond vitamin D: integrating nuclear receptor signaling networks in periodontal health and disease

**DOI:** 10.3389/fphys.2026.1969566

**Published:** 2026-09-29

**Authors:** Yujia Chen, Liancheng Guan, Qian Li, Yihui Chai, Jie Gao

**Affiliations:** 1 The Second Affiliated Hospital of Guizhou University of Traditional Chinese Medicine, Guiyang, Guizhou, China; 2 Qihuang College of Guizhou University of Traditional Chinese Medicine, Guiyang, Guizhou, China

**Keywords:** FXR, heterodimerization, nuclear receptor signaling, periodontal disease, PPAR-γ, RXR-α, VDR

## Abstract

Vitamin D receptor (VDR) signaling has been studied extensively in periodontology, yet the broader nuclear receptor superfamily remains underexplored. We examined the roles of four nuclear receptor family members: VDR, peroxisome proliferator-activated receptor gamma (PPAR-γ), retinoid X receptor alpha (RXR-α), and farnesoid X receptor (FXR) in periodontal health and disease across the continuum from gingival health through gingivitis to periodontitis. However, most functional and mechanistic evidence is derived from established or experimental periodontitis, whereas evidence from periodontal health and gingivitis remains limited. VDR, PPAR-γ, and FXR have been associated with periodontal immune and bone responses through distinct but intersecting pathways. VDR induces cathelicidin and modulates RANKL/OPG expression. PPAR-γ suppresses NF-κB-mediated cytokine production and promotes M2 macrophage polarization. FXR agonists reduce osteoclastogenesis while enhancing osteoblast differentiation in rodent periodontitis models. In general nuclear receptor biology, RXR-α is a heterodimerization partner for VDR, PPAR-γ, and FXR and may represent a putative integrative node in periodontal signaling. However, the direction and magnitude of RXR-α expression changes remain inconsistent, and the effects of RXR ligand activation on VDR and PPAR-γ transcriptional activity in periodontal tissues remain to be established. Major gaps remain: human data on PPAR-γ and FXR are scarce, localized nuclear receptor activation has not advanced to clinical trials, and nuclear receptor interactions with periodontal biofilms remain uncharacterized. A network-centered framework prioritizing RXR-mediated heterodimerization, localized ligand delivery, nuclear receptor-selective modulators, and genotype-guided supplementation may help define future research directions, but these applications require further validation.

## 1 Introduction

Periodontal diseases rank among the most prevalent chronic inflammatory conditions worldwide, affecting the tissues that surround and support the teeth. Recent global burden estimates based on the 2021 Global Burden of Disease study place the total number of prevalent periodontal disease cases at approximately 1.07 billion, with 89.6 million new incident cases annually (Hu et al., 2025). These figures aggregate a heterogeneous spectrum of clinical states rather than a single disease entity. The 2017 World Workshop on the Classification of Periodontal and Peri-Implant Diseases and Conditions distinguishes periodontal health, gingivitis, and periodontitis as interrelated but separable conditions (Chapple et al., 2018). Gingivitis confined to the gingival soft tissues produces reversible inflammation and resolves after plaque removal (Trombelli, 2025). Periodontitis extends to the periodontal ligament and alveolar bone, causing clinical attachment loss and leaving permanent damage even when inflammation is controlled (Könönen et al., 2019).

The economic and clinical burden of periodontal disease is not limited to advanced periodontitis (Kassebaum et al., 2014; Listl et al., 2015). Gingivitis accounts for a substantial share of dental visits and oral health expenditure, while even low-grade gingival inflammation has been linked to systemic health outcomes (Sanz et al., 2020; Botelho et al., 2022). The efficacy of conventional treatments is often compromised by the recurrence of plaque and gingivitis, as well as the continued advancement of periodontitis (Tonetti et al., 2015). This persistent therapeutic challenge has driven research toward new targeted adjuncts capable of fortifying periodontal support structures in the earlier stages of the disease spectrum (de Molon et al., 2026).

Nutritional and endocrine factors modulate susceptibility across the entire periodontal continuum (Lu, 2023). Vitamin D status, for example, correlates with gingival inflammation in population-based studies and has been tested as an adjunct to non-surgical therapy for gingivitis (Dietrich et al., 2005). In a randomized controlled trial, daily supplementation with 500, 1000, or 2000 IU of vitamin D^3^ for three months was associated with dose-related reductions in gingival index scores compared with placebo, with the earliest and greatest improvement observed in the 2000 IU/day group (Srivastava et al., 2023). At the cellular level, vitamin D induces the antimicrobial peptide LL-37 and the innate immune receptor TREM-1 in gingival epithelial cells, responses that strengthen epithelial barrier defense before attachment loss occurs (McMahon et al., 2011). Thus, VDR signaling is relevant during gingival inflammation, not only after bone destruction has begun. VDR belongs to a nuclear receptor superfamily of 48 ligand-activated transcription factors (Germain et al., 2006b). Several family members are expressed in gingival and periodontal tissues across health and disease states. PPAR-γ, a regulator of macrophage polarization and lipid metabolism, has been detected in gingival tissues from healthy individuals, patients with periodontitis, and patients with peri-implantitis (Taskan and Gevrek, 2020). PPAR-γ, RXR-α, and VDR have been evaluated in human gingival tissues from periodontally healthy individuals and patients with periodontal disease. Disease-associated differences in their expression have been reported, although the direction and statistical significance of these changes vary across studies (Evans and Mangelsdorf, 2014; Taskan and Gevrek, 2020; Karatas et al., 2021). A recent systematic review and meta-analysis found lower PPAR-γ protein levels in periodontal tissues from experimental periodontitis models, whereas the limited human studies showed inconsistent findings (Hidalgo et al., 2025). In general nuclear receptor biology, RXR-α serves as a heterodimerization partner for VDR and PPAR-γ; however, these complexes have not been directly demonstrated in periodontal tissues. Its expression has been reported to change in periodontitis compared with healthy controls, although the direction and magnitude of this change remain inconsistent (Taskan and Gevrek, 2020). FXR, a bile acid sensor, has only recently been investigated in preclinical periodontitis models (Huang et al., 2024). These receptors are therefore present throughout the periodontal continuum, but most mechanistic studies have focused on periodontitis endpoints such as bone loss, leaving their roles in gingivitis and periodontal health comparatively unexplored (Altucci et al., 2007).

The coexistence of these receptors within the same periodontal microenvironments raises a question that has not been systematically addressed across the full disease spectrum. Do these nuclear receptors function as isolated pathways, or do they constitute an integrated signaling network? In other tissues, RXR forms heterodimers with VDR, PPAR-γ, and FXR, and ligand activation of RXR can potentiate or repress the transcriptional activity of its partners depending on cellular context (Rőszer et al., 2013). Whether simultaneous modulation of multiple nuclear receptors offers advantages over single-receptor targeting remains unknown and requires direct evaluation in relevant periodontal models. The question becomes especially relevant for gingivitis, where tissue destruction is still absent and modulation of epithelial innate immunity and macrophage polarization could prevent progression (Leal et al., 2023).

We synthesize evidence for VDR, PPAR-γ, RXR-α, and FXR across periodontal health and disease, examine their individual roles from gingival inflammation to periodontitis, and evaluate the rationale for a proposed RXR-centered network. These four receptors were selected because they represent the most relevant available links to antimicrobial defense, inflammation, lipid or bile-acid signaling, bone remodeling, and nuclear receptor heterodimerization. We interpret these pathways within a contemporary framework involving dysbiotic biofilms, host–microbe interactions, epithelial barrier dysfunction, inflammation resolution, and osteoimmunology. Within this framework, the receptors discussed in this review may influence distinct but intersecting biological checkpoints rather than act as isolated anti-inflammatory pathways. To define the scope of the evidence discussed in this review, we searched PubMed/MEDLINE, Web of Science Core Collection, and Scopus from database inception through July 2026, using combinations of periodontal terms such as “periodontitis,” “gingivitis,” and “periodontal tissue” with nuclear receptor terms including “VDR,” “PPAR-γ,” “RXR-α,” and “FXR.” We included peer-reviewed human, animal, and periodontal-cell studies addressing receptor expression, function, genetic associations, or targeted interventions. Non-periodontal studies were used only as clearly identified mechanistic context. Evidence from chronic apical periodontitis was considered separately from plaque-associated marginal periodontitis.

## 2 Nuclear receptor signaling mechanisms in periodontal tissues

### 2.1 VDR: the best-characterized periodontal nuclear receptor

VDR mediates most biological effects of the vitamin D endocrine system. It is a 50-kDa nuclear transcription factor in the nuclear receptor subfamily 1 (NR1I1). Upon binding 1,25-dihydroxyvitamin D3 (1,25(OH)^2^D^3^), VDR heterodimerizes with RXR and binds to vitamin D response elements (VDREs) in target gene promoters, modulating transcription of genes involved in calcium homeostasis, antimicrobial defense, and immune regulation (Núñez et al., 2010; Haussler et al., 2011).

VDR expression has been documented across multiple cell types in periodontal tissues (Wang et al., 2004; Lu, 2023; He et al., 2025). Immunofluorescence studies of human periodontal ligament cells (hPDLCs) have confirmed nuclear VDR protein expression, which is upregulated by exogenous 1,25(OH)^2^D^3^ in a concentration-dependent manner (Gao et al., 2018). Gingival fibroblasts similarly express VDR, and treatment with 1,25(OH)^2^D^3^ has been reported to induce cathelicidin antimicrobial peptide (CAMP/LL-37) expression in these cells, suggesting a potential role for VDR signaling in periodontal antimicrobial defense (Wang et al., 2004; Lu, 2023; He et al., 2025). In gingival epithelial cells, vitamin D-related signaling may contribute to antimicrobial barrier defense (Liu et al., 2006; McMahon et al., 2011). However, direct evidence that VDR activation restores epithelial barrier integrity or reverses dysbiosis in human periodontal disease remains limited (Hajishengallis and Lamont, 2021; Lu, 2023).

Vitamin D-related signaling has also been investigated in the osteogenic differentiation of periodontal ligament stem cells (PDLSCs). *In vitro* studies have reported that 1,25(OH)^2^D^3^ promotes or supports osteogenic differentiation and osteogenic-marker expression in hPDLSCs under selected experimental conditions, including inflammatory conditions (Ji et al., 2019; Zhang et al., 2020; Qi et al., 2021). In a rat model of ligature-induced periodontitis, vitamin D deficiency was associated with greater clinical attachment loss and radiographic alveolar bone loss, whereas vitamin D-supplemented animals exhibited less severe periodontal destruction (Zainal et al., 2023).

Beyond its genomic effects, VDR also exerts non-genomic actions via a membrane-associated isoform (mVDR) that activates protein kinase C (PKC) and mitogen-activated protein kinase (MAPK) signaling within minutes of ligand binding. The contribution of non-genomic VDR signaling to periodontal homeostasis remains largely unexplored (Adams and Hewison, 2008).

### 2.2 PPAR-γ: an anti-inflammatory nuclear receptor with equivocal periodontal evidence

PPAR-γ was originally characterized as a regulator of adipogenesis and insulin sensitivity, but it is now recognized as a potent anti-inflammatory transcription factor that antagonizes NF-κB signaling (Chawla et al., 2001; Wagner and Wagner, 2020). PPAR-γ activation suppresses pro-inflammatory cytokine production, including TNF-α, IL-1β, and IL-6, and promotes anti-inflammatory macrophage phenotypes in experimental or non-periodontal systems (Ricote et al., 1998; Daynes and Jones, 2002; Szanto and Nagy, 2008). These mechanisms may be relevant to the resolution of periodontal inflammation, but they should not be interpreted as evidence of a consistent change in PPAR-γ expression or function in human periodontitis. Thus, PPAR-γ may participate in inflammation resolution, particularly through macrophage-state transition, although its contribution to resolution in human periodontal lesions has not been directly established (Ricote et al., 1998; Croasdell et al., 2015).

The expression and functional relevance of PPAR-γ in periodontal tissues have been systematically reviewed in 2025. Hidalgo-Caldeira et al. conducted a meta-analysis of six studies (four experimental, two clinical) and reported that healthy animals exhibit higher PPAR-γ protein levels in periodontal tissues compared with animals with experimental periodontitis (Z = 3.07, p = 0.002) (Hidalgo et al., 2025). However, the two human immunohistochemical studies reported contradictory findings: Taskan and Gevrek observed higher PPAR-γ expression in gingival tissues from patients with periodontitis (Taskan and Gevrek, 2020), whereas Karatas et al. reported the opposite pattern (Karatas et al., 2021). Their pooled analysis showed no significant difference (Z = 0.03, p = 0.97), with extreme heterogeneity (I^2^ = 100%) (Hidalgo et al., 2025).

Several factors may account for this discrepancy. Differences in tissue collection and processing, the inflammatory status of sampled tissues, and the anatomical site of harvest—such as gingival margin versus alveolar mucosa—could each contribute to inter-study variability. Furthermore, PPAR-γ expression is highly responsive to local microenvironmental cues, including the profile of microbial challenge and the presence of dietary ligands (e.g., omega-3 fatty acids, which are endogenous PPAR-γ agonists). These complexities underscore the need for standardized methodologies in future clinical investigations of PPAR-γ in periodontitis (Di Paola et al., 2006; Villacorta et al., 2009; Lan et al., 2023; Wang et al., 2025; Zhou et al., 2026).

### 2.3 RXR-α: a proposed integrative node of nuclear receptor signaling

RXR-α occupies a unique position within the nuclear receptor superfamily. Unlike many nuclear receptors that can form both homodimers and heterodimers, RXR serves as an obligate heterodimerization partner for roughly one-third of all nuclear receptors, including VDR, PPAR-γ, FXR, and the thyroid hormone receptor. The functional consequence is that RXR ligand binding can, depending on the specific heterodimer and cellular context, either potentiate or inhibit the transcriptional activity of its partner (Altucci et al., 2007; Rőszer et al., 2013; Leal et al., 2023).

In periodontal tissues, co-expression studies have assessed RXR-α protein alongside VDR and PPAR-γ in gingival tissues from chronic periodontitis patients and healthy controls. RXR-α expression is altered in diseased periodontal tissues, though the direction and magnitude of change vary across reports. In periodontal tissues, RXR-α, VDR, and PPAR-γ have been evaluated concurrently in human gingival samples, and disease-associated differences in their expression patterns have been reported (Taskan and Gevrek, 2020; Karatas et al., 2021; Karatas and Gevrek, 2026). However, concurrent detection does not establish receptor–receptor association, physical heterodimerization, coordinated transcriptional activity, or mutual stabilization.

This observation has direct therapeutic implications. If RXR expression or ligand availability were limiting factors for partner-receptor activity in periodontal tissues, pharmacological activation of RXR might theoretically modulate the transcriptional activity of permissive heterodimers, including RXR–PPARγ, in selected cellular contexts. This possibility is supported primarily by general nuclear-receptor pharmacology and non-periodontal studies showing that RXR agonists can alter heterodimer-dependent transcription and immune responses (Altucci et al., 2007; Rőszer et al., 2013; Evans and Mangelsdorf, 2014; Leal et al., 2023). However, direct RXR–VDR, RXR–PPARγ, or RXR–FXR functional interactions have not been demonstrated in periodontal tissues. The target engagement, local pharmacokinetics, efficacy, dose requirements, and local and systemic safety of RXR-directed therapy in periodontal disease therefore remain unknown.

### 2.4 FXR: a nascent periodontal nuclear receptor target

FXR (NR1H4) functions as an intracellular bile acid sensor and regulates metabolic homeostasis, inflammatory responses, and mucosal barrier integrity (Wang et al., 2008; Modica et al., 2010). Although FXR is best known for its roles in hepatic and intestinal physiology, recent evidence indicates that FXR signaling is active in oral tissues. Huang et al. (2024) demonstrated that FXR agonist treatment attenuates alveolar bone loss and gingival inflammation in a rat ligature-induced periodontitis model. The therapeutic effects were accompanied by suppressed NF-κB p65 nuclear translocation, reduced TLR2 and TLR4 expression, and upregulated Runx2 and Ibsp expression in periodontal tissues (Huang et al., 2024).

The multitarget potential of FXR signaling is of interest for periodontal applications. In periodontal cell and rat ligature-induced periodontitis models, FXR agonist treatment reduced pro-inflammatory signaling and osteoclastogenesis and increased the expression of osteoblast-related markers, including RUNX2 and IBSP (Huang et al., 2024). In non-periodontal bone models, FXR activation has also been reported to promote osteoblast differentiation and suppress osteoclast differentiation (Cho et al., 2013). Whether these effects translate to human periodontitis remains to be determined, as no clinical investigation of FXR ligands in periodontal disease has been reported to date.

## 3 RXR-centered heterodimerization: the networking logic of nuclear receptor

The preceding section describes four nuclear receptors that may influence periodontal pathophysiology. VDR, PPAR-γ, and RXR-α have been detected in human gingival tissues, whereas the available periodontal evidence for FXR is derived primarily from cell and rodent models (Taskan and Gevrek, 2020; Karatas et al., 2021; Huang et al., 2024). Outside the periodontal context, structural and functional studies have demonstrated ligand- and coregulator-dependent signaling within VDR–RXR, PPARγ–RXR, and FXR–RXR heterodimers (Orlov et al., 2012; Kojetin et al., 2015; Wang et al., 2018). These findings provide a mechanistic rationale for proposing an RXR-centered integrative framework in periodontal tissues.

RXR may represent a potential integrative node within the proposed conceptual framework. As established in general nuclear receptor biology, RXR forms heterodimers with multiple nuclear receptors, including VDR, PPAR-γ, and FXR (Lefebvre et al., 2010; Evans and Mangelsdorf, 2014). Structural studies have demonstrated receptor-specific interactions and allosteric communication within VDR–RXR, PPARγ–RXR, and FXR–RXR heterodimers (Orlov et al., 2012; Kojetin et al., 2015; Wang et al., 2018). The ligand-response properties of these heterodimers differ. PPARγ–RXR is generally classified as permissive because ligands targeting either receptor can activate or enhance transcription, whereas VDR–RXR is conventionally classified as non-permissive, although the response to RXR ligands may vary with promoter architecture, ligand combination, coregulator availability, and cellular context (Germain et al., 2006a; Kojetin et al., 2015). These mechanistic distinctions may be relevant to therapeutic design. An RXR agonist could theoretically influence multiple nuclear receptor pathways in selected cellular contexts; however, direct target engagement, transcriptional effects, efficacy, and safety have not been established in periodontal tissues (Altucci et al., 2007; Leal et al., 2023).

The periodontal relevance of RXR-centered heterodimerization is currently supported only indirectly by receptor-expression studies. VDR, PPAR-γ, and RXR-α have been evaluated concurrently in human gingival tissue samples, and disease-associated differences in their expression patterns have been reported across periodontally healthy individuals and patients with periodontitis (Taskan and Gevrek, 2020; Karatas and Gevrek, 2026). However, concurrent detection within the same tissue does not demonstrate receptor–receptor association, physical heterodimerization, coordinated transcriptional activity, or mutual stabilization. We identified no periodontal study directly demonstrating VDR–RXR, PPARγ–RXR, or FXR–RXR complex formation using co-immunoprecipitation, proximity ligation, chromatin immunoprecipitation, or comparable interaction assays. Direct demonstration of these complexes in periodontal tissues or periodontal cell models therefore remains an important experimental gap.

A related question concerns the potential for nuclear receptor crosstalk at the level of target-gene regulation. VDR and PPAR-γ regulate distinct but partially overlapping inflammatory pathways. In non-periodontal experimental systems, VDR activation has been reported to reduce NF-κB DNA binding and NF-κB-dependent transcription, whereas PPAR-γ can antagonize NF-κB-regulated genes through several context-dependent mechanisms, including competition for transcriptional coactivators and SUMOylation-dependent retention of NCoR–HDAC3 corepressor complexes at inflammatory gene promoters (Harant et al., 1997; Li et al., 2000; Pascual et al., 2005). These mechanistic observations provide a rationale for investigating whether combined VDR and PPAR-γ activation produces additive, synergistic, or antagonistic effects; however, such interactions cannot be inferred from their individual anti-inflammatory activities. Systematic investigations of nuclear receptor combinatorial effects in periodontal cells are, to our knowledge, absent from the current literature. As shown in **Figure 1**, the proposed RXR-centered framework integrates VDR–RXR, PPAR-γ–RXR, and FXR–RXR interactions while distinguishing established mechanisms from hypotheses requiring validation.

**Figure 1:**
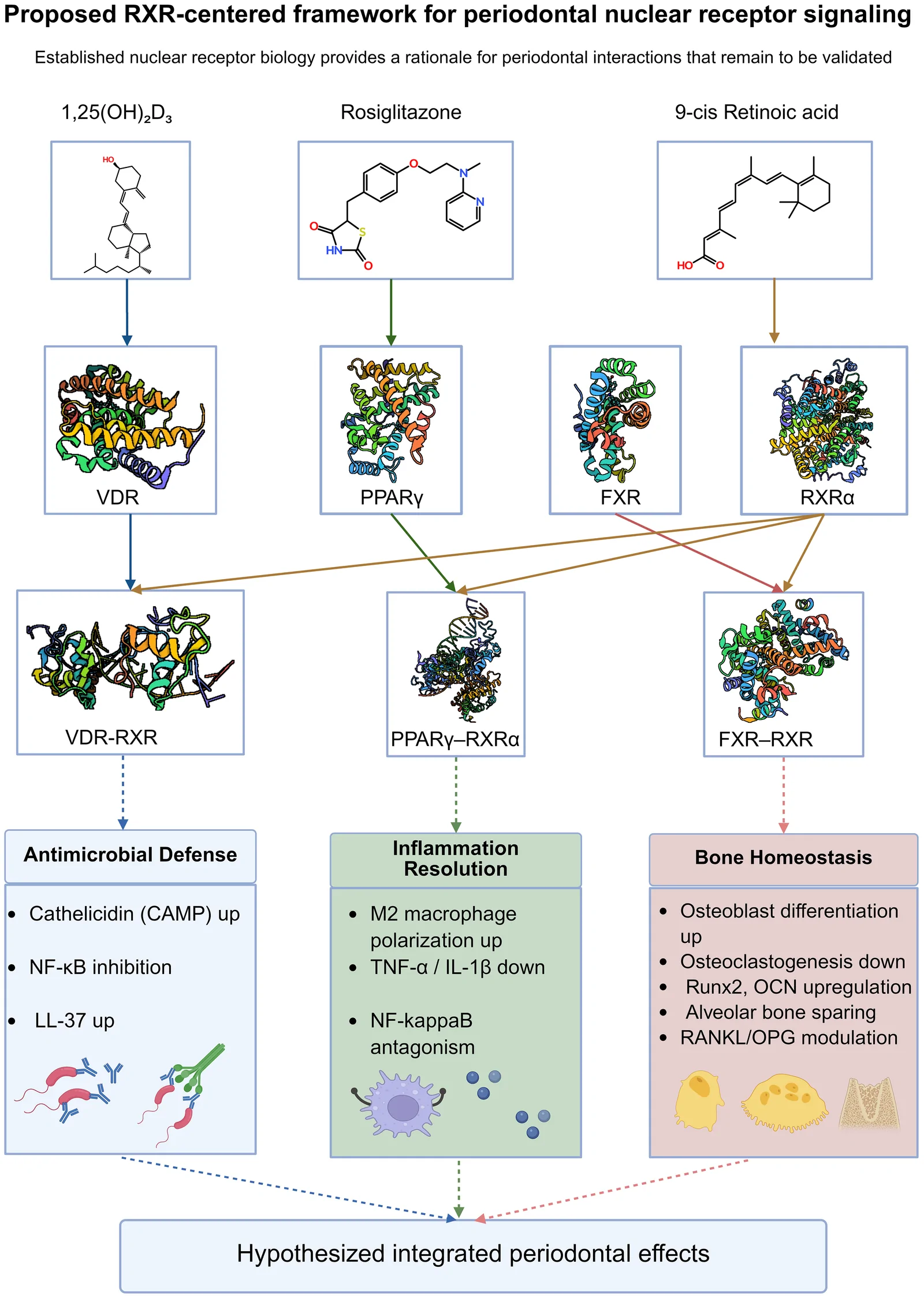
Proposed RXR-centered conceptual framework for nuclear receptor signaling in periodontal tissues. VDR, PPAR-γ, RXR-α, and FXR may influence antimicrobial defense, inflammation resolution, and bone remodeling through distinct but potentially intersecting pathways. The depicted receptor interactions and downstream outcomes are proposed mechanisms based primarily on general nuclear receptor biology and non-periodontal evidence. The structural models illustrate general nuclear receptor biology and do not represent experimentally confirmed complexes in periodontal tissues.

## 4 Genetic susceptibility: nuclear receptor polymorphisms and periodontal disease risk

Genetic variation in nuclear receptor genes may modify periodontal disease susceptibility by altering receptor expression, ligand binding affinity, or transactivation capacity. The most extensively studied nuclear receptor genetic association in periodontitis involves the VDR gene, which harbors several commonly studied polymorphisms.

### 4.1 VDR polymorphisms

Four VDR polymorphisms have been frequently investigated in relation to periodontitis: FokI (rs2228570), located in the translation initiation region; TaqI (rs731236), located near the 3′ end of the gene; and BsmI (rs1544410) and ApaI (rs7975232), located in the intronic region near the 3′ end of VDR. The FokI polymorphism alters the use of alternative translation initiation sites, with the F allele producing a VDR protein that is three amino acids shorter than that produced by the f allele. Although one experimental study reported greater transcriptional activity of the shorter F-VDR isoform, another found no significant differences in ligand affinity, DNA binding, or transcriptional activation between the two isoforms (Arai et al., 1997; Gross et al., 1998). By contrast, BsmI, ApaI, and TaqI are generally considered noncoding or synonymous marker polymorphisms without consistently established direct functional effects. Associations involving these variants may reflect linkage disequilibrium with other functional variants in the VDR 3′ region rather than direct effects on VDR expression, mRNA stability, or splicing (Uitterlinden et al., 2004).

A meta-analysis of 38 case-control studies investigating VDR polymorphisms and chronic periodontitis risk reported no statistically significant association for BsmI, TaqI, FokI, or ApaI in the overall analyses. Ethnicity-stratified analyses identified isolated nominal associations, including an association between the BsmI A allele and periodontitis risk among Caucasian participants (OR = 1.747, 95% CI: 1.099–2.778; 1 study; p = 0.018) and an inverse association for the ApaI recessive model (TT vs. TG + GG) in a mixed-population subgroup (OR = 0.488, 95% CI: 0.239–0.996; 1 study; p = 0.049). Because both subgroup estimates were based on single studies, they should be interpreted cautiously and do not contradict the null overall findings (Mashhadiabbas et al., 2018). The differences between the overall and ethnicity-stratified estimates may reflect variation in linkage disequilibrium patterns, gene–environment interactions, including sunlight exposure and baseline vitamin D status, and the genetic backgrounds of the populations studied (Koromantzos et al., 2012; Cocate et al., 2019; Woelber et al., 2023; Dragičević Tomičić et al., 2025).

The clinical translation of VDR polymorphism findings remains uncertain. No interventional study has, to our knowledge, stratified patients by VDR genotype before assigning vitamin D supplementation, leaving open the question of whether VDR genotype modifies treatment response. This represents a tractable and important question for future periodontal nutrition trials.

### 4.2 PPAR-γ and RXR polymorphisms

In contrast to the voluminous VDR polymorphism literature, genetic associations between PPAR-γ or RXR gene variants and periodontitis risk have scarcely been investigated. Two small case-control studies have examined PPAR-γ Pro12Ala (rs1801282) in relation to periodontitis, with conflicting results. The scarcity of data precludes any firm conclusion regarding whether PPAR-γ genetic variation influences periodontal disease susceptibility (Hirano et al., 2010; Wang et al., 2016). No eligible study examining RXR gene polymorphisms and periodontitis susceptibility was identified within the scope of this review.

This asymmetry in investigation effort is puzzling, given that PPAR-γ and RXR are at least as well positioned as VDR to influence periodontal inflammation. One possible explanation is that the VDR–periodontitis research trajectory was established early, creating a citation bias that perpetuates VDR-focused investigations while marginalizing other nuclear receptors. Whatever the reason, the near-absence of PPAR-γ and RXR genetic studies in periodontitis constitutes a notable evidence gap. The available genetic association evidence for nuclear receptor polymorphisms and periodontitis risk, including overall and ethnicity-specific estimates, is summarized in **Table 1**. The pooled estimates for the four VDR polymorphisms were derived from the systematic review and meta-analysis by Mashhadiabbas et al. (2018), whereas the PPAR-γ and RXR rows indicate the limited or absent evidence identified within the scope of this review.

**Table 1:** Overall and ethnicity-specific associations between VDR polymorphisms and periodontitis risk, with limited evidence for PPAR-γ and RXR.

NR Gene	SNP	Analysis level and genetic model	OR (95% CI)	Population; studies; cases/controls	P value	I^2^ (%)
VDR	BsmI (rs1544410)	Overall; A vs. G	1.159 (0.896–1.498)	All populations; 9 studies; 866/786	0.261	52.98
VDR	BsmI (rs1544410)	Ethnicity-specific subgroup; A vs. G	1.747 (1.099–2.778)	Caucasian; 1 study; 72/102	0.018	NA
VDR	TaqI (rs731236)	Overall; C vs. T	0.946 (0.828–1.080)	All populations; 16 studies; 1570/1676	0.408	82.28
VDR	FokI (rs2228570)	Overall; T vs. C	1.051 (0.842–1.311)	All populations; 5 studies; 374/382	0.660	0.00
VDR	ApaI (rs7975232)	Overall; T vs. G	0.737 (0.469–1.157)	All populations; 7 studies; 632/604	0.184	84.45
VDR	ApaI (rs7975232)	Ethnicity-specific subgroup; TT vs. TG+GG	0.488 (0.239–0.996)	Mixed; 1 study; 110/50	0.049	NA
PPAR-γ	Pro12Ala (rs1801282)	—	—	—	Insufficient data	—
RXR	—	—	—	—	No eligible association study identified within the search scope	—

The overall analyses of all four VDR polymorphisms were not statistically significant. The BsmI and ApaI estimates from ethnicity-specific analyses were based on single studies and should therefore be interpreted cautiously. I^2^ was not estimable for ethnicity-specific analyses based on a single study and is reported as NA.

## 5 Therapeutic implications: from systemic supplementation to localized nuclear receptor activation

### 5.1 Vitamin D supplementation as adjunctive periodontal therapy

Observational studies and systematic reviews have reported lower vitamin D concentrations or a higher frequency of vitamin D insufficiency among some populations with periodontitis, although the direction and strength of this association vary across studies (Isola et al., 2020; Machado et al., 2020). Preclinical periodontal studies have also reported that vitamin D-related interventions can modulate inflammation, antimicrobial responses, and alveolar bone loss in cell and animal models (Menzel et al., 2019; Kirkwood et al., 2024). Completed randomized clinical trials have evaluated vitamin D supplementation as an adjunct to scaling and root planing (SRP), but the reported clinical benefits have been modest and have varied according to baseline vitamin D status, supplementation regimen, follow-up duration, and study design (Gao et al., 2020; Perić et al., 2020).

An updated systematic review and meta-analysis published in 2023 included 16 studies involving 1,455 participants and evaluated both the association between vitamin D status and periodontitis and the effects of vitamin D supplementation. In the adjunctive-treatment analysis, only three studies contributed data to the meta-analysis of clinical attachment level (CAL) and probing depth (PD). The pooled result showed a small improvement in CAL favoring vitamin D supplementation combined with scaling and root planing (WMD = −0.13 mm, 95% CI: −0.19 to −0.06), whereas the pooled reduction in PD was not statistically significant (WMD = −0.11 mm, 95% CI: −0.23 to 0.01; p = 0.07) (Liang et al., 2023). Given the small number of contributing intervention studies and the magnitude of the estimated CAL difference, the clinical relevance and generalizability of these findings remain uncertain.

Dose-response relationships have not been systematically characterized in periodontal clinical trials; direct comparative evidence that ≥4000 IU/day provides greater periodontal benefit is currently lacking. The available evidence on nuclear receptor ligands, experimental models, principal outcomes, and translational limitations is summarized in **Table 2**.

**Table 2:** Nuclear receptor ligands and periodontal relevance: experimental models, evidence bases, principal outcomes, evidence levels, limitations, and supporting references.

Receptor	Ligand/intervention	Model/condition	Evidence base	Principal outcome	Evidence level	Major limitation	Supporting references
VDR	1,25(OH)^2^D^3^; vitamin D supplementation	Human cohorts; periodontal cells; rodents; SRP trials	Observational studies, cell studies, animal models, and small RCTs	Antimicrobial and osteogenic responses; small CAL improvement signal	Human association + preclinical + limited clinical	Baseline status, dose, and follow-up heterogeneous	(McMahon et al., 2011; Gao et al., 2018; Ji et al., 2019; Menzel et al., 2019; Gao et al., 2020; Isola et al., 2020; Machado et al., 2020; Perić et al., 2020; Zhang et al., 2020; Qi et al., 2021; Liang et al., 2023; Zainal et al., 2023; Kirkwood et al., 2024)
PPAR-γ	Rosiglitazone and other PPAR-γ agonists	Human gingival tissues; macrophage/cell systems; rat periodontitis	Small human expression studies and predominantly preclinical functional studies	Anti-inflammatory effects; conflicting human expression findings	Preclinical + inconsistent human evidence	Context- and ligand-dependent; no periodontal clinical trial	(Ricote et al., 1998; Di Paola et al., 2006; Taskan and Gevrek, 2020; Karatas et al., 2021; Hidalgo et al., 2025; Karatas and Gevrek, 2026; Zhou et al., 2026)
RXR-α	9-cis-retinoic acid; RXR-selective agonists proposed	Human gingival expression; non-periodontal heterodimer biology	Small human expression studies plus non-periodontal mechanistic evidence	Disease-associated expression differences; periodontal heterodimerization unproven	Indirect/limited periodontal evidence	No direct periodontal complex formation or target-engagement evidence	(Germain et al., 2006a; Altucci et al., 2007; Lefebvre et al., 2010; Orlov et al., 2012; Rőszer et al., 2013; Evans and Mangelsdorf, 2014; Kojetin et al., 2015; Wang et al., 2018; Taskan and Gevrek, 2020; Karatas et al., 2021; Leal et al., 2023; Karatas and Gevrek, 2026)
FXR	FXR agonists; bile-acid-related ligands	Periodontal cells; rat ligature periodontitis; non-periodontal bone models	Periodontal-cell and rodent evidence only	Inflammatory, osteoclast, and osteoblast-related effects reported	Preclinical only	No periodontal clinical study identified	(Wang et al., 2008; Modica et al., 2010; Cho et al., 2013; Wang et al., 2018; Huang et al., 2024)

### 5.2 Localized nuclear receptor activation: a paradigm shift

The renal production of circulating 1,25(OH)^2^D^3^ is subject to homeostatic regulation. In the kidney, CYP27B1 converts 25(OH)D to 1,25(OH)^2^D^3^ and is stimulated by parathyroid hormone but inhibited by FGF23, high calcium and phosphate concentrations, and feedback from 1,25(OH)^2^D^3^; CYP24A1 contributes to vitamin D metabolite catabolism (Bikle, 2014). Excessive or prolonged high-dose vitamin D intake can increase the risk of hypercalcemia and hypercalciuria, although clinically significant vitamin D toxicity is uncommon under appropriately monitored supplementation (Malihi et al., 2016; Tebben et al., 2016). However, the relationship between systemic vitamin D dose and the concentration of active vitamin D metabolites within human periodontal tissues has not been established.

A 2024 study investigated the topical application of inactive vitamin D^3^ in mice with ligature-induced periodontitis. Daily intraoral administration of vitamin D^3^ reduced alveolar bone loss, local inflammation, and osteoclast formation and was associated with changes in the oral microbial community. Serum 25(OH)D^3^ concentrations did not differ significantly among the treatment groups, suggesting limited systemic exposure under the experimental conditions. However, the study did not directly establish CYP27B1-dependent local conversion within periodontal tissues, assess serum calcium concentrations, or demonstrate safety and efficacy in humans (Kirkwood et al., 2024).

This localized activation strategy is, in principle, generalizable to other nuclear receptors. RXR ligands, PPAR-γ agonists, or FXR agonists could similarly be formulated for local delivery to periodontal pockets using gels, nanoparticles, or controlled-release strips. Potential advantages include higher local ligand concentrations at the disease site and potentially reduced systemic exposure. However, local delivery does not eliminate the possibility of local or systemic adverse effects, and the safety and efficacy of combinatorial nuclear receptor activation remain to be established.

### 5.3 Nuclear receptor selective modulators: precision targeting

Therapeutically desirable nuclear receptor ligand properties differ depending on the clinical objective. For periodontal bone regeneration, a VDR-selective agonist that promotes osteoblast differentiation without causing hypercalcemia would be ideal. For periodontal inflammation control, a PPAR-γ-selective agonist that suppresses NF-κB signaling without inducing adipogenesis (a known off-target effect of PPAR-γ full agonists) would be preferable. For comprehensive periodontal protection, an RXR-selective agonist could theoretically influence multiple nuclear receptor pathways, although its effects would depend on receptor abundance, heterodimer permissiveness, ligand dose, inflammatory context, and disease stage. No nuclear receptor-selective modulator has been clinically tested for periodontal indications. The development of such agents may represent a potential translational opportunity, but their efficacy and safety require evaluation in relevant preclinical periodontal models.

The therapeutic performance of nuclear receptor ligands will depend not only on their biological activity but also on target engagement, tissue exposure, pharmacokinetics, receptor selectivity, and disease context. Ligand efficacy may vary according to receptor abundance, cellular composition, heterodimer permissiveness, inflammatory status, and disease stage. Moreover, activation of one receptor may alter the activity of other nuclear receptor heterodimers or induce broader transcriptional changes, creating potential off-target effects. Full PPAR-γ agonists have been associated with adipogenic and metabolic effects, including weight gain and edema, whereas selective PPAR-γ modulators are being investigated as a means of retaining anti-inflammatory activity while reducing these liabilities (Zhang et al., 2007). RXR agonists may influence multiple RXR-containing heterodimers and produce broad, context-dependent transcriptional responses; the clinical pharmacology of bexarotene also illustrates potential systemic metabolic and endocrine adverse effects (Duvic et al., 2001; Altucci et al., 2007; Rőszer et al., 2013; Leal et al., 2023). FXR-directed agents may affect bile-acid and hepatic pathways beyond periodontal tissues and may be associated with treatment-related adverse effects such as pruritus (Wang et al., 2008; Cho et al., 2013; Smith and Pegram, 2017). These observations derive mainly from non-periodontal pharmacology and should not be interpreted as evidence of toxicity in periodontal applications. Local delivery could increase drug exposure at periodontal sites and potentially reduce systemic exposure, but it does not establish favorable local pharmacokinetics or eliminate local toxicity, systemic absorption, or off-target transcriptional effects. Therefore, dose optimization, receptor selectivity, tissue distribution, pharmacokinetic profiling, target engagement, and both local and systemic safety should be established in clinically relevant periodontal models before therapeutic translation.

## 6 Research gaps and future directions

Several gaps must be closed before nuclear receptor signaling advances can be translated into clinical periodontal applications. First, direct evidence for nuclear receptor network integration in human periodontal tissues is lacking. Co-immunoprecipitation studies confirming VDR-RXR and PPARγ-RXR heterodimer formation in human gingival or periodontal ligament cells have not been reported. Chromatin immunoprecipitation (ChIP) sequencing of VDR, PPAR-γ, and RXR in periodontal cells under inflammatory conditions could define the nuclear receptor target gene repertoire and reveal whether nuclear receptor crosstalk occurs at shared DNA response elements. Second, the temporal dynamics of nuclear receptor signaling during periodontal disease initiation and progression remain unknown. Whether nuclear receptor signaling dysfunction precedes clinical periodontitis or results from established inflammation is unclear. Longitudinal studies measuring nuclear receptor expression in gingival crevicular fluid or biopsied tissues before and after periodontitis onset would clarify the causal role of nuclear receptor signaling in human periodontal disease. Third, nuclear receptor microbiome interactions have not been investigated. The 2024 topical vitamin D^3^ study reported altered subgingival microbial composition after local VDR activation, raising the possibility that nuclear receptor signaling influences and is influenced by the periodontal microbiome. Given the established role of FXR in regulating gut microbiota composition, a similar nuclear receptor microbiome axis in periodontal tissues is plausible and warrants investigation. Fourth, human clinical evidence for PPAR-γ and FXR targeting is essentially absent. The 2025 systematic review of PPAR-γ in periodontitis identified only two small clinical studies with conflicting results, and no clinical study of FXR ligands in periodontitis has been reported. Further studies should first establish target engagement, local pharmacokinetics, dose–response relationships, efficacy, and safety in clinically relevant preclinical periodontal models before early-phase clinical evaluation is considered. The clinical relevance of VDR genetic variation also remains uncertain. Whether VDR genotype predicts the response to vitamin D supplementation is unknown, because no genotype-stratified intervention trial has established such an effect. After appropriate clinical and functional validation, exploratory genotype-stratified trials could test whether VDR genotype modifies treatment response. As illustrated in **Figure 2**, topical vitamin D^3^ delivery currently represents a preclinical proof-of-concept, whereas localized RXR-, PPAR-γ-, and FXR-ligand delivery remains unvalidated.

**Figure 2:**
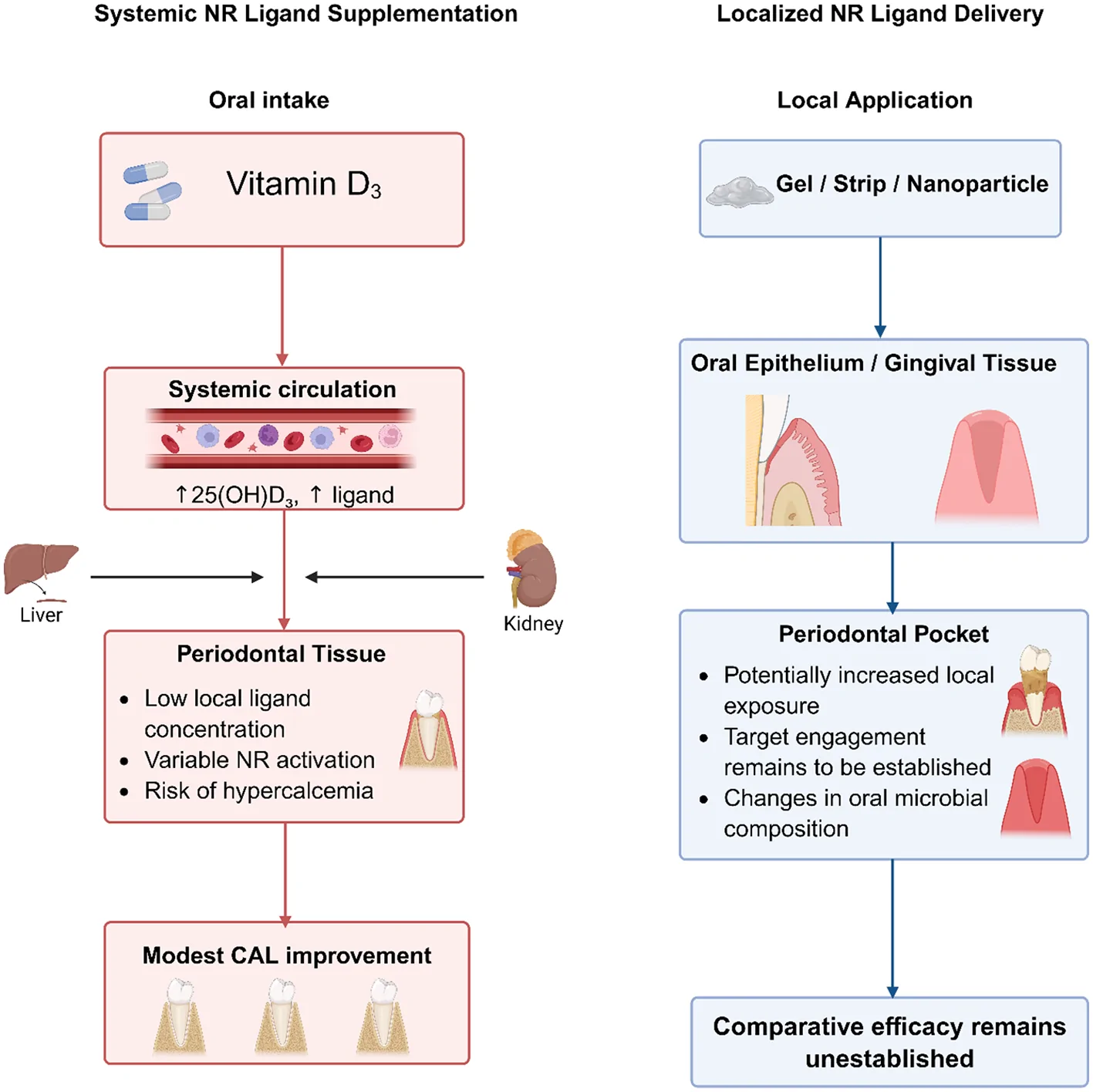
Conceptual comparison of systemic and localized nuclear receptor ligand delivery in periodontal tissues. The depicted differences in ligand exposure, receptor activation, microbial responses, and clinical effects are hypothetical. Topical vitamin D^3^ has shown preclinical effects in experimental periodontitis, whereas localized delivery of RXR, PPAR-γ, and FXR ligands remains investigational.

## 7 Conclusions

Nuclear receptor signaling networks remain an underappreciated dimension of periodontal pathobiology. The available evidence, although fragmentary, supports a proposed framework in which VDR, PPAR-γ, RXR-α, and FXR may influence periodontal tissue responses through independent and potentially intersecting pathways. RXR-centered heterodimerization provides a plausible molecular basis for the proposed integration of nuclear receptor signaling; however, direct experimental validation in human periodontal tissues is still needed.

The translational implications of these findings are scientifically plausible but remain preliminary. Localized nuclear receptor activation may increase ligand exposure at periodontal sites while potentially reducing systemic exposure; however, target engagement, local pharmacokinetics, receptor selectivity, off-target transcriptional effects, dose optimization, local and systemic safety, and clinical efficacy remain to be established. Nuclear receptor-selective modulators validated for periodontal indications might enable more precise targeting of individual receptor pathways, but their therapeutic benefits and risks require further evaluation. Personalized VDR genotype-guided supplementation should be considered a long-term research hypothesis rather than an established therapeutic strategy.

Moving beyond vitamin D as a solitary focus broadens the mechanistic and therapeutic landscape of periodontal nuclear receptor research. The evidence assembled here supports further investigation of receptor-specific and network-level mechanisms while emphasizing the need for rigorous validation in periodontal models and clinical studies.
